# First results of a refeeding program in a psychiatric intensive care unit for patients with extreme anorexia nervosa

**DOI:** 10.1186/s12888-015-0436-7

**Published:** 2015-03-24

**Authors:** Christoph Born, Larissa de la Fontaine, Bettina Winter, Norbert Müller, Annette Schaub, Clemens Früstück, Cornelius Schüle, Ulrich Voderholzer, Ulrich Cuntz, Peter Falkai, Eva Meisenzahl

**Affiliations:** 1 Department of Psychiatry, Ludwig Maximilians-University, Nussbaumstrasse. 7, 80336 Munich, Germany; 2 Schoen-Klinik Roseneck, Prien am Chiemsee, Germany

**Keywords:** Eating disorders, Anorexia nervosa, Refeeding, Percutaneous gastric feeding tube, High caloric solution

## Abstract

**Background:**

Anorexia nervosa (AN) is associated with a high mortality rate. This study describes a compulsory re-feeding program established in Munich for extremely underweight patients.

**Methods:**

The contract between the patient and the therapeutic team included mandatory inpatient status, establishment of guardianship and compulsory re-feeding with a percutaneous gastric feeding tube, as indicated. The predefined target was a body mass index (BMI) of 17 kg/m^2^. Data on the first 68 patients with AN are presented.

**Results:**

65 (95.6%) patients were female and mean age at admission was 26.5 ± 8.5 years. BMI increased from 12.3 ± 1.4 kg/m^2^ at admission to 16.7 ± 1.7 kg/m^2^ at discharge. Thirty-two (47.1%) patients had the restrictive subtype (ANR) and 36 (52.9%) had the binging and purging subtype (ANBP). Duration of illness before admission (p = .004), days of treatment until discharge (p = .001) and weight increase (p = .02) were significantly different between subgroups in favor of patients with ANR. Also, seasonal differences could be found. Comparison of feeding methods showed that percutaneous tube feeding was superior. Almost half of the patients were treated with psychotropic medication. To date, however, the number of patients included in this program is too small to assess rare complications of this acute treatment program and long term outcomes of AN.

**Conclusions:**

An intensive care program for severely ill AN patients has been successfully established. Besides averting physical harm in the short term, this program was designed to enable these patients to participate in more sophisticated psychotherapeutic programs afterwards. To our knowledge, this is the first such program that regularly uses percutaneous feeding tubes.

## Background

Anorexia nervosa (AN) is associated with one of the highest mortality rates in psychiatry. For example, a Swedish register study comparing observed with expected death rates reported that people hospitalized for AN had a standardized mortality rate of 6.2 [1], and a follow-up study of 51 patients hospitalized for AN found that only half were symptom-free after 18 years [2]. Although the incidence of AN has been reported to have increased in the last few decades [3], others have reported that this perception is incorrect, with apparent increases being due to complex interrelations among age, observational period, and cohort effects [4,5]. However, an epidemiological study in adolescents found symptoms of eating disorders in 24% of girls and 16% of boys [6].

Epidemiological assessments of morbidity and mortality have shown that AN is one of the most serious disorders in psychiatry [7]. Its psychiatric and somatic co-morbidities indicate that AN is complex and difficult to treat. Many co-morbid psychiatric disorders have been detected in patients with eating disorders. For example, the lifetime prevalence rates of mood disorders were estimated to be as high as 75% in patients with eating disorders, and the co-morbidity rates of obsessive-compulsive disorder, anxiety disorders and substance abuse to be 40%, 11–20%, and 40%, respectively [8]. Adolescents with an affective disorder were reported to be at higher risk of developing an eating disorder [9]. Personality traits often found in patients with AN include perfectionism, impulsivity and obsessive-compulsive characteristics. Obsessive-compulsive personality disorder is the most common personality disorder in patients with restrictive AN (ANR), whereas borderline personality disorder is the most prominent in patients with the binging and purging subtype of AN (ANBP) [10].

Malnutrition and long-time starvation affect almost every organ in the body [11]. Cardiac complications are observed in about 80% of these patients; one of the most threatening consequences is atrophy of the heart, leading to alterations in cardiac electrical activity, structure and hemodynamics. Atrophy may result in secondary prolongation of the QT-interval and cardiac arrhythmia, complications predictive of sudden cardiac death [12]. Reduced concentrations of sodium and potassium contribute not only to cardiac arrhythmia, but may lead to myelinolysis of the pons [13]. Serum concentrations of phosphate and magnesium are often diminished during enforced refeeding, which may contribute to the development of a ‘refeeding syndrome’ [14,15]. The most frequent endocrine dysfunction associated with AN is amenorrhea [16], although chronic starvation may induce type 1 diabetes mellitus [17].

Due to the potentially lethal impact of AN on physical health, and studies showing that weight restoration significantly ameliorated several neuropsychological aspects of AN [18,19] and reduced EEG abnormalities [20], the first goal of AN treatment should be restoration of weight. A detailed program for compulsory re-feeding of patients with severe AN has therefore been established over the last decade at the Department of Psychiatry, University Hospital of Munich. This report describes the demographic characteristics and short term outcomes of the first 68 AN patients enrolled in this program.

## Methods

This study investigated a subsample of approximately 100 patients with AN and severe underweight who had been admitted to our hospital between 2000 and 2013. Some patients were admitted two or three times because of relapse. All patients included in this analysis were admitted for the first time to our psychiatric intensive care unit (PICU) and were treated according to the formalized re-feeding program. The local ethics board of the Ludwig Maximilians-University consented to this evaluation of prospectively collected, routine clinical data for scientific purposes.

The re-feeding program had been developed especially for patients with AN and extreme underweight, as described [21]. Of the 68 included patients, 51 (75%) had a body mass index (BMI) under 13 kg/m^2^ at admission. Based on British (NICE, Eating Disorders, Clinical guidelines no. 9, 2004, www.nice.org.uk [22]) and German (Diagnostik und Therapie der Essstörungen, S3-Leitlinie der AWMF, 2010, www.awmf.org [23]) guidelines for the inpatient treatment of AN, the primary aim of the refeeding program was to attain a BMI of 17 kg/m^2^ by gaining 700–1000 g per week. Cornerstones of the program include legal guardianship, acceptance of a percutaneous gastric feeding tube if not contraindicated for medical reasons, and a detailed contract between the patient and the therapeutic team; this contract couples steps of weight gain with leave from the ward, reduction of feeding with high caloric nutrients and participation in additional therapeutic programs and psychotherapy.

Legal guardianship of these patients was established according to Bavarian state law. If the responsible medical officer decided that a patient admitted to hospital lacks the insight or capacity to make informed decisions on medical treatment and need for hospitalization to avoid acute harm to self or others, that officer could apply for sectioning and the granting of legal guardianship at a local court. A section and legal guardianship had been established in almost all of our patients before or at the time of admission. The section consisted of the right to administer compulsory treatment, including enforced feeding if necessary, and inpatient status with restrictions of free movement for a fixed time period.

Besides offering all patients regular meals, they were advised to undergo insertion of a percutaneous gastric feeding tube, unless there were medical contraindications. Based on treatment targets, most patients had to gain approximately 10 kg of body weight to reach 90% of the ideal body weight within an acceptable time frame. Feeding with a percutaneous gastric tube was chosen for several reasons. First, additional feeding using a tube was likely to be necessary for several months. In contrast, prolonged use of a nasogastric tube can cause damage to the nose or upper gastrointestinal tract and the tube has to be changed regularly. Second, patients are more handicapped when consuming food orally. Third, patients were more likely to manipulate or remove a nasogastric than a percutaneous gastric tube. Finally, a nasogastric tube can be stigmatizing in a PICU environment, which also admits patients with other psychiatric disorders.

The contract between the patient and the treatment team was regarded as a basic tool of behavioral therapy to motivate the patient. This contract included rules of behavior, types of therapy being offered, rewards and restrictions.

At the beginning of the program, psychotherapy was offered only upon demand and only for management of an acute crisis. Regular psychotherapeutic sessions were offered when patients reached a BMI of approximately 15 kg/m^2^ and were considered able to participate in regular sessions of 50 minutes each. Participation in therapeutic programs should not interfere with the primary aim of gaining body weight. Moreover, most underweight patients feel restless owing to their mental and physical condition, with this restlessness making participation in psychotherapeutic sessions problematic.

All AN patients were required to participate in common dining on the ward. Feeding with a high caloric solution was considered an additional substitute treatment, as most of these patients were unable to finish their regular meals because of compensatory distracting behaviors, overvalued ideas, gastrointestinal discomfort, and other reasons. Following implantation of a feeding tube, a high caloric solution was titrated individually, to a maximum of 3000 kcal per day. In the following weeks the amount of the high caloric solution was adjusted, targeting a weight gain of 700–1000 g per week. If necessary, patients could be treated with psychotropic medications to target psychiatric syndromes such as depression and lack of weight gain. Vitamins, electrolytes, and other nutrients were administered to compensate for current deficits.

The program mandated that high caloric solution be stopped after patients reached a BMI of approximately 17 kg/m^2^, and that the feeding tube be removed if body weight remained stable for 2 weeks. Future therapy was planned, on an in- or outpatient basis, with most patients selecting eating disorder specific psychotherapy at a specialized hospital. Most of these patients had been referred from these hospitals to our PICU when they were extremely underweight.

Weight gain was analyzed in the intention-to-treat (ITT) population using the last observation carried forward (LOCF) method. Categorical variables were reported as frequencies and compared using the chi-square test, and continuous variables were reported as mean ± standard deviation (SD) and compared using t tests. Comparisons of patients before and after treatment were assessed using paired t tests.

## Results

The analysis includes 68 consecutively admitted patients who were treated for the first time for AN on our ward and agreed to participate in the program. Of these, 65 (95.6%) were female and 3 (4.4%) were male; 32 (47.1%) were diagnosed with ANR and 36 (52.9%) with ANBP. Interestingly, all three male patients were diagnosed with ANR. At admission the mean age of the 68 patients was 26.5 years (SD 8.5 years; range 16–61 years) and their mean duration of AN was 9.5 years (SD 6.8 years; range 1–33 years). Mean treatment in the PICU was 150.2 days (SD 80.8 days; range 56–348 days). Mean BMI was 12.3 kg/m^2^ (SD 1.4 kg/m^2^; range 8.8–14.9 kg/m^2^) at admission and 16.7 kg/m^2^ (SD 1.7 kg/m^2^; range 11–21.2 kg/m^2^) at discharge. Mean weight increase was 12.0 kg (SD 5.1 kg; range −0.2–20.3 kg).

Following admission, 57 (83.8%) patients underwent implantation of a percutaneous gastric feeding tube, three (4.4%) underwent implantation of a nasogastric tube and eight (11.8%) received no feeding tube because of contraindications (e.g., ascites). Thirty-four (50%) patients were referred from psychotherapeutic in- or outpatient settings, from which they did not benefit because of overvalued ideas, delusions or extensive compensatory behavior.

A comparison of the demographic characteristics and treatment outcomes of patients in the ANR and ANBP subgroups showed that duration of illness until admission to our hospital (p = .005) and days of treatment until discharge from our specialized unit (p = .001) were significantly shorter and total weight gain significantly higher (p = .02) in the ANR than in the ANBP subgroup (Table 1).

**Table 1:** **Demographic and clinical characteristics of patients with ANR and ANBP**

	ANR	ANBP	P
**Patients (n)**	32 (47.1%)	36 (52.9%)	
**Female (n)**	29 (90.6%)	36 (100%)	
**Age (years)**	25.6 ± 10.0	27.3 ± 7.0	p = .4*
**Duration of illness (years)**	7.0 ± 5.7	11.6 ± 7.1	p = .004*
**Days of treatment**	118.0 ± 57.2	178.9 ± 88.4	p = .001*
**BMI at admission**	11.9 ± 1.4	12.6 ± 1.3	p = .06*
**BMI at discharge**	16.9 ± 1.6	16.4 ± 1.8	p = .3*
**Weight increase (kg)**	13.5 ± 3,7	10.7 ± 5.7	p = .02*
**Medication at discharge (n)**	17 (53.1%)	23 (63.9%)	p = .09**
**Antidepressants**	16 (50%)	21 (58.3%)	p = .15**
**Antipsychotics**	6 (18.8%)	9 (25%)	p = .1**
**Anticonvulsants**	0 (0%)	3 (8.3%)	-
**L-thyroxine**	3 (9.4%)	6 (16.6%)	p = .4**

*t test, **chi square test.

*Abbreviations: ANR* Anorexia nervosa, restrictive subtype, *ANBP* Anorexia nervosa, binging/purging subtype.

**Figure 1:**
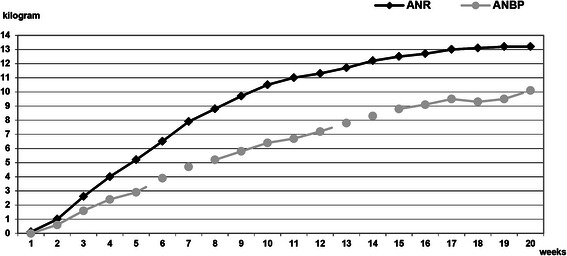
**Course of weight gain (kg/week) in the ANR and ANBP diagnostic subgroups during the 20 weeks after admission.** Abbreviations: ANR, anorexia nervosa, restrictive subtype; ANBP, anorexia nervosa, binging/purging subtype.

A comparison of the course of weight gain in the two subgroups over the first 20 weeks after admission showed that the kinetics of weight gain were higher in the ANR subgroup, suggesting that binging and purging in the ANBP group may have suppressed weight gain (Figure 1).

Seasonal differences in BMI at admission were compared in patients in the ANR and ANBP subgroups admitted during autumn and winter with those admitted during spring and summer. More total patients (57.3% vs. 42.7%), as well as patients in the ANR (59.4% vs. 40.6%) and ANBP (55.6% vs. 44.4%) subgroups, were admitted during autumn and winter than during spring and summer. A comparison of ANR and ANBP patients admitted during autumn and winter showed that mean BMI (11.8 ± 1.6 kg/m^2^ vs. 12.8 ± 1.3 kg/m^2^; p = .04) and days of treatment until discharge (120.6 ± 54.3 days vs. 191.2 ± 84.5 days; p = .003) were significantly lower in the ANR than in the ANBP subgroup, whereas there were no differences in mean BMI and days of treatment between ANR and ANBP patients admitted during spring and summer (p > .1 each).

**Figure 2:**
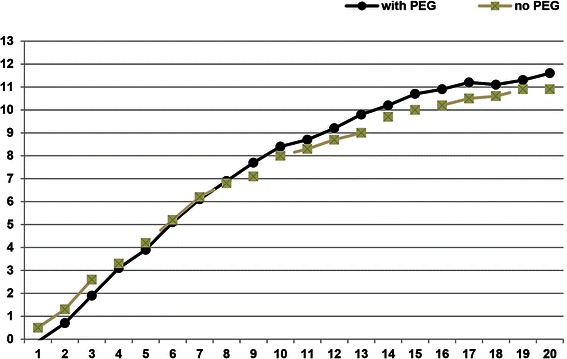
**Course of weight gain over 20 weeks in groups of patients with and without percutaneous feeding tubes (PEG).**

A comparison of the 57 patients with and the 11 without a percutaneous feeding tube showed that mean duration of treatment was significantly longer in patients with percutaneous feeding tubes (p < .01). However, patients without a percutaneous feeding tube were younger (mean age 22.9 ± 6.1 vs. 27.2 ± 8.8 years), had a shorter mean duration of illness (7.3 ± 5.5 vs. 9.9 ± 7.0 years) and a slightly higher mean BMI (12.7 ± 1.3 vs. 12.2 ± 1.4 kg/m^2^) at admission, and lower mean total weight gain (11.3 ± 4.7 vs. 12.2 ± 5.2 kg, n.s.) than patients with percutaneous feeding tubes. Figure 2 illustrates the course of weight gain over 20 weeks in patients with and without percutaneous gastric feeding tubes.

Examination of blood chemistry in these patients at admission and before discharge showed no significant changes in red and white blood cell counts, and in sodium and potassium concentrations (Table 2). Blood chemistry findings did not differ significantly in the ANR and ANBP subgroups (data not shown).

**Table 2:** **Blood chemistry and ECG results at admission and at discharge**

Chemistry	Admission (n = 68)	Discharge (n = 68)
Leucopenia (<4.0 G/l)	26 (38.2%)	19 (27.9%)
Anemia (RBC, Hb, Hc below normal range)	19 (27.9%)	18 (26.5%)
Hyponatremia (<135 mmol/l)	18 (26.5%)	10 (14.7%)
Hypokalemia (<3.5 mmol/l)	19 (27.9%)	12 (17.7%)
**ECG**	1st ECG (n = 43)	Last ECG (n = 43)
Days after admission	5.1 ± 6.4	124.8 ± 86.4
Bradycardia (<50 BPM)	8 (18.4%)	3 (7.0%)
Tachycardia (>100 BPM)	2 (4.7%)	4 (9.3%)
BPM (mean ± SD)	61.1 ± 17.0	73.7 ± 15.9

*Abbreviations: G/l* 10^9^ cells per liter, *RBC* red blood cells, *Hb* hemoglobin, *Hc* hematocrit, *BPM* beats per minute, *SD* standard deviation.

Of the 68 patients, 43 (63.2%) underwent ECG during the first 25 days (mean 5.1 ± 6.4 days) after admission. ECGs showed bradycardia, defined as a heart rate < 50/min, in eight patients (18.4%) and tachycardia, defined as a heart rate ≥100/min, in two (4.7%). ECGs performed before discharge in these patients, at a mean 124.8 ± 86.4 days after admission, showed bradycardia in three patients (7.0%) and tachycardia in four (9.3%). Mean heart rate increased significantly, from 61.1 ± 17.0 bpm to 73.7 ± 15.9 bpm (two sided *t* test; p < .001).

Of the 52 patients (76.5%) who underwent cerebral MRI, three (5.8%) had central pontine myelinolysis and one (1.9%) had signs of brain atrophy. Bone density was measured at the lumbar part of the vertebral column and the femur in 27 patients (39.7%). Of these, 19 (70.4%) had osteoporosis, with mean femoral and lumbar mineral contents of 0.75 ± 0.16 g/cm^2^ and 0.86 ± 0.16 g/m^2^, respectively; and eight (29.6%) had osteopenia, with mean femoral and lumbar mineral contents of 0.81 ± 0.18 g/cm^2^ and lumbar 0.91 ± 0.11 g/cm^2^, respectively. Only one patient (3.7%) had a normal mineral content.

Psychotropic medication was offered to patients with any comorbid psychiatric illness requiring treatment, such as depression, generalized anxiety, or obsessive-compulsive disorder, but not with the intention to affect weight. Almost two thirds of these patients were being treated with psychotropic medication at the time of discharge (Table 1). Thirty-seven patients (54.4%) received various antidepressants as monotherapy (imipramine, trimipramine, citalopram, escitalopram, fluoxetine, paroxetine, sertraline, mirtazapine, reboxetine or venlafaxine), and five (7.4%) received a combination of two antidepressants (SSRI + mirtazapine). Fifteen patients (22.1%) received antipsychotics (clozapine, olanzapine, perazine, quetiapine, risperidone or zotepine). Additionally, all 68 patients were being treated with vitamins or other nutritional substitutes. No significant differences in medication use were observed between the ANR and ANBP subgroups.

Patients who attained a BMI of approximately 17 kg/m^2^ were actively encouraged to arrange for further therapy. Patients with ANBP were more likely to seek further treatment in a psychiatric or psychotherapeutic hospital (n = 30; 83.3%) than patients with ANR (n = 18; 56.3%), whereas patients with ANR more often favored further treatment in an outpatient setting (37.5% vs. 13.9%).

## Discussion and conclusions

Treatment settings and offers for people with AN and extreme underweight (BMI <13 kg/m^2^) are scarce. The described detailed treatment protocol in a PICU setting is potentially lifesaving for these patients. To our knowledge, this is the only program that routinely includes the use of a percutaneous gastric feeding tube, emphasizing the severity of AN and its resistance to treatment. Most of the included patients were referred from psychotherapeutic settings after failure of less restrictive approaches.

The results reported in this article indicate that patients with severe AN can be successfully treated, at least by reversing a life threatening condition. Clearly, this does not necessarily mean a lasting change of mind set in these patients. At admission, most of the patients had a BMI under 13 kg/m^2^, achieving a BMI of 16 to 17 kg/m^2^ at discharge. Weight gain was easier to achieve in patients with ANR. However, patients with ANBP had a significantly longer mean duration of illness (11.1 vs. 6.3 years; p < .004), which may indicate a more severe and chronic course of illness.

Differences in BMI at admission and days of treatment in subgroups of patients with ANR and ANBP were found to be seasonal because more patients were admitted during autumn and winter than during spring and summer [24]. We also found that BMI at admission (p = .04) and days of treatment until discharge (p = .003) differed significantly in patients with ANR and ANBP admitted during autumn and winter (p = .04), but not during spring and summer (p > .1 each).

The results of comparisons of patients with and without percutaneous gastric feeding tubes must be interpreted with caution because of the small sample size of the latter. We found that weight gain was slightly higher in patients with a percutaneous tube. Moreover, slight but not significant differences from admission to discharge were observed in the blood chemistry of patients with percutaneous tubes. This may have been due to unchanged compensatory behavior (e.g. vomiting) or hormonal recovery (e.g. onset of menstrual function) rather than to re-feeding.

This study had several limitations, including its retrospective design and the uncontrolled, naturalistic nature of the results. Patients were not randomized to treatment regimens. The retrospective design explains missing data for ECG, MRI and measurements of bone mineral density, as those examinations were not part of a prospective study protocol. Of the patients that underwent bone density measures, 93.7% were diagnosed with osteoporosis or osteopenia, indicating the severe physical sequelae of AN in our patients.

Different strategies are used to treat patients with AN. Disorder specific psychotherapy is considered first line treatment [25,26]. Pharmacotherapy trials designed to increase appetite and ameliorate the constricted and obsessive thinking in AN, including those with olanzapine or quetiapine, have yielded inconsistent results [27,28]. A recent meta-analysis showed that weight gain was significantly affected by hormonal therapy, but not by treatment with antidepressants or antipsychotics, compared with placebo [29]. Finally, use of a feeding tube and high caloric solution, may be the only successful lifesaving treatment strategy for the most severely undernourished patients with AN.

A Medline search using the terms “eating disorders”, “anorexia nervosa” and “refeeding” identified eight articles published in the last decade. Most were retrospective analyses of the outcomes of refeeding programs for patients with AN [30-36], with outcomes defined as recovery and complication rates. Only one prospective, randomized study compared oral and nasogastric administration of a high caloric solution [37]. In six studies, use of a nasogastric tube was compared with oral refeeding, one compared oral refeeding with a high caloric solution versus oral refeeding with normal food [33], and one compared parenteral and oral refeeding [30]. Two of these studies included only adolescent patients [30,35], and the duration of refeeding varied considerably, from 15.6 days [30] to 12.3 ± 1.9 weeks [33]. In addition, body weights at baseline and endpoint varied widely. In three studies, body weights at start and after refeeding were comparable to those in our patients [33,34,37]. These three studies included only adult patients, but the observation period varied from 56 days [37] to 12.3 ± 1.9 weeks [33]. Only one of these studies included a standardized follow-up at 3 and 12 months after discharge, with the time to recovery significantly longer in the group using nasogastric feeding tubes (p < .05) [37]. Another study reported that, after reaching a mean BMI of 13.5 ± 1 kg/m^2^, 29 patients continued therapy in an outpatient setting, finally achieving a mean BMI of 18.4 ± 2.3 kg/m^2^, but the duration of follow-up was not reported [33]. Although the methods and results of these studies were quite heterogeneous, all found that administration of a high caloric solution using a feeding tube was superior to oral refeeding.

Little is known about long term outcomes after refeeding programs. A mean 13.5-year follow-up of 484 adult patients with AN found that 60.3% had fully recovered, 25.8% had what was described as a good outcome, 6.4% a bad outcome, 6.4% had a severe outcome, and 1.2% had died [38]. Binge-eating/purging subtype and personality disorder were identified as predictors of an inferior outcome [38]. A comparison of 6-year outcomes in 41 severely malnourished AN patients (mean BMI at admission 10.1 ± 0.57 kg/m^2^), started immediately on tube-refeeding, and 443 less malnourished AN patients reported death rates of 7% and 1.2%, respectively; severe outcome rates of 29% and 10%, respectively; and recovery rates of 41% and 62%, respectively. This indicated that prudent tube refeeding, especially in AN patients with a BMI <11 kg/m^2^, could reduce short-term mortality rates, although long-term prognosis remains poor [39]. Although long term data are not available for our cohort, only seven (10.3%) were readmitted for re-feeding.

The program described in this article was established for the most severely affected AN patients who are at risk of death or irreversible physical damage [21]. As normalization of body weight can also restore cognitive function [19,40], these patients may be able to participate more successfully in psychotherapeutic programs.

## Abbreviations

AN: Anorexia nervosa
ANBP: Anorexia nervosa, binging/purging subtype
ANR: Anorexia nervosa, restrictive subtype
AWMF: Arbeitsgemeinschaften der wissenschaftlichen medizinischen Fachgesellschaften
BMI: Body mass index
BPM: Beats per minute
ECG: Electrocardiography
G/l: 10^9^ cells per liter
Hb: Hemoglobin
Hc: Hematocrit
ITT: Intention-to-treat
LOCF: Last observation carried forward
MRI: Magnetic resonance imaging
NICE: National Institute for Health and Care Excellence
PEG: Percutaneous gastric feeding tube
PICU: Psychiatric intensive care unit
RBC: Red blood cells
SD: Standard deviation
